# An Aggressive Juvenile Nasopharyngeal Angiofibroma With Intracranial and Orbital Extension in an Adolescent Female: A Case Report and Focused Literature Review

**DOI:** 10.7759/cureus.90338

**Published:** 2025-08-17

**Authors:** Muhammad Ayub, Sobia Ahmed, Quang Dai La, Alisa Bryantseva, Aiman Baloch, Hafsa Qayyum, Muhammad Ashraf Kasi, Francis Pryor

**Affiliations:** 1 Radiology, Bolan Medical Complex Hospital, Quetta, PAK; 2 Biology, Texas A&M University, College Station, USA; 3 Medicine, The Innovative STEMagazine, College Station, USA; 4 Biomedical Engineering, Johns Hopkins University, Baltimore, USA; 5 Medicine, Mekran Medical College, Turbat, PAK; 6 Radiology, Modern X-rays and Imaging Centre, Quetta, PAK; 7 Medicine, Lake Erie College of Osteopathic Medicine, Erie, USA

**Keywords:** adolescent, female patient, intracranial extension, juvenile nasopharyngeal angiofibroma, orbital invasion, skull base tumor, sphenopalatine foramen, vascular tumor

## Abstract

Juvenile nasopharyngeal angiofibroma (JNA) is a rare, benign but locally invasive vascular tumor of childhood that almost exclusively affects adolescent males. We present a very unusual case of a 14-year-old female with JNA, who presented with persistent headache, nasal obstruction, post-nasal drip, and intermittent epistaxis. Contrast-enhanced CT imaging revealed a highly enhancing mass located in the sphenopalatine fossa, coursing into the left nasal cavity, nearby soft tissues, paranasal sinuses, and eroding the hard palate. Additional imaging showed that the mass extended into the orbit and intracranium, occupying the left middle cranial fossa. The patient underwent multidisciplinary surgical management by otorhinolaryngologists and neurosurgeons.

This case contributes to the sparse literature on JNA in genetically female patients. Although rare female cases have been reported, most angiofibromas in females have occurred post-menopause or have arisen from atypical extra-nasopharyngeal locations. This instance is unique in that we demonstrate a classic nasopharyngeal origin in an adolescent female, which adds to the current literature on the atypical population demographics of JNA. A review of past female cases further supports the conclusion that physicians should include JNA in the differential diagnosis for vascular nasopharyngeal masses, regardless of sex.

## Introduction

Juvenile nasopharyngeal angiofibroma (JNA) is a rare, benign, yet locally aggressive vascular tumor, accounting for approximately 0.05% of all head and neck neoplasms [1,2]. It typically originates from the superior margin of the nasopharynx, specifically involving the sphenopalatine foramen, vidian canal, or pterygopalatine fossa - an anatomically complex junction where branches of the maxillary artery and neural crest-derived tissues converge [3]. Juvenile angiofibromas usually arise in adolescent males (ages 10-20 years) and rarely occur in females (12.5% of all cases) [1].

Patients typically present with unilateral nasal obstruction and epistaxis, among other symptoms, which can include headache, facial swelling, rhinorrhea, and anosmia, based on the size and extent of the tumor [1]. Radiologically, these tumors appear as remarkably enhancing masses that localize to the sphenopalatine foramen, with bony remodeling and typical routes of spread, including the paranasal sinuses, orbits, and intracranially [4].

The pathogenesis of JNA is still not completely understood, though hormonal and molecular mechanisms appear to be important. Androgen receptors are found in JNA tissue, suggesting a possible androgen-dependent tumorigenesis. Studies have shown that hormone receptor pathways (such as the androgen receptor) and growth factors (such as VEGF and TGF-β) may also play a role in tumor development [5].

Approximately 4%-20% of JNA cases report intracranial extension, most frequently as extradural extension, including the middle cranial fossa or cavernous sinus, and sometimes orbital involvement [1]. Previous reports indicate that management of JNA, particularly with skull base or intracranial involvement, is challenging due to the neoplasm’s hypervascularity and local invasion characteristics. Surgical excision is the cornerstone of treatment, typically following preoperative embolization to control intraoperative bleeding [6].

Open surgical techniques have a long history of description - midfacial degloving, transpalatal, infratemporal fossa, craniofacial, and others - for tumors extending significantly [7]. The newer concepts of expanded endoscopic endonasal approaches, combined with limited external access, offer the advantage of decreased morbidity and better visualization, and have established themselves to the point of being our choice for many cases, even some with moderate intracranial involvement [8]. Tumors that invade intradurally or extensively invade the cavernous sinus may require combined or staged approaches, including craniotomy with transfacial access, and in select cases, adjuvant radiotherapy may still be under consideration [9].

This report describes a relatively rare case of JNA in a 14-year‑old female, with significant intra‑orbital and intracranial extension, which made surgical management difficult. It touches on the role of imaging in detailing the extent of the tumor and the need for surgeons to work in a multidisciplinary manner.

This case was presented at the 40th Annual RSP Radiological Conference, in collaboration with the Royal College of Radiologists (UK), on November 8-10, 2024, at the Pearl Continental Hotel, Rawalpindi, Pakistan.

## Case presentation

A 14-year-old female patient presented to the ENT clinic with complaints of persistent headache, increasingly troublesome sinonasal congestion, and postnasal drip with occasional nosebleeds. The clinical assessment of the patient included contrast-enhanced CT imaging of the sinuses.

**Figure 1:**
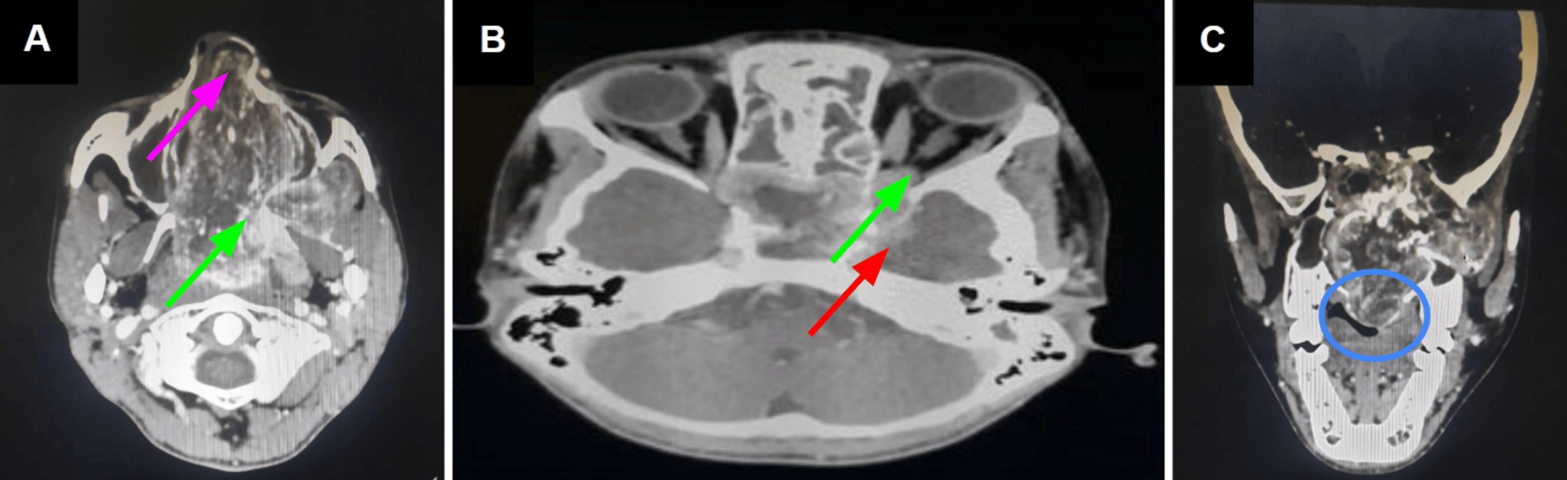
Contrast enhanced computed tomography axial (A & B) and coronal (C) images. (A) An avidly enhancing mass is noted in the nasopharyngeal region, centered in the left sphenopalatine foramen, causing its widening (green arrow) and extending towards the right side. The main bulk is noted in the left nasal cavity (pink arrow). (B) Intraorbital (green arrow) and intracranial (red arrow) extension into the left middle cranial fossa was also noted, as evident by the enhancing component. (C) It is extending into adjacent soft tissues and also causing erosion of bony walls. Invasion of the paranasal sinuses was also evident, with involvement of the hard palate (blue circle).

Imaging revealed a mass with avid enhancement starting from the sphenopalatine fossa, causing widening of this space and having its main bulk extending into the left nasal cavity (Figure 1A). Further imaging evaluation showed intra-orbital extension as well as intracranial extension into the left middle cranial fossa, demonstrated by the enhancing intracranial component (Figure 1B). The mass also had extension into the surrounding soft tissues with bony erosion, including involvement of the paranasal sinuses and hard palate (Figure 1C).

The patient was managed by both the ENT and neurosurgery teams, given the findings from the imaging.

## Discussion

JNA is a highly vascular, benign tumor of the anterior skull base that overwhelmingly affects adolescent males. Female patients with nasopharyngeal angiofibroma are exceedingly rare - large case series (120 cases) have reported no females [10]. Reported exceptions span a wide age range: Gruber et al. described a seven-year-old female in 1985 [11], and Ralli et al. recently reported a 68-year-old woman (the oldest on record) [12]. These rare female cases include a few adolescent females, as well as several post-menopausal women. Notably, most angiofibromas in females have been either in older patients or have originated in atypical sites outside the nasopharynx [13].

It is important to distinguish extra-nasopharyngeal angiofibromas (ENPA) - tumors arising in the nasal cavity or sinuses rather than the nasopharyngeal sphenopalatine area - from JNA. ENPAs are considered to be clinically distinct: they tend to occur at a slightly older age and disproportionately affect women [14]. For example, angiofibromas arising from the nasal septum, ethmoid, or turbinate have been reported in female patients across various ages [13]. In one case of a 16-year-old girl with an ethmoid sinus angiofibroma, karyotyping was performed to rule out any Y-chromosome material and confirmed a normal female genotype [15]. However, the present case involves a classic nasopharyngeal angiofibroma in an adolescent female - unusually rare compared to the overwhelmingly male prevalence of JNA. 

Several individual case reports in the literature mirror aspects of our case. For instance, Finerman described a 13-year-old female with a nasopharyngeal angiofibroma (managed with radiotherapy) as one of the earliest female cases on record [16]. Osborn and Sokolovski reported a 15-year-old girl with a nasopharyngeal angiofibroma that recurred after surgery [17]. Sporadic reports continued over the years, affirming that, while rare, JNA can indeed occur in genetically female patients. On the other end of the spectrum, post-menopausal women have presented with angiofibromas in the nasopharynx. Recently, Ralli et al. described a 68-year-old female patient, highlighting that both female sex and advanced age made their case exceptionally uncommon [12]. 

The most immediately comparable report is the 2022 account by Sikdar et al., who followed an 11-year-old female treated for JNA and a decade later documented her unprecedented complication of internal carotid artery canal dehiscence [13].

## Conclusions

This case supports the observation that JNA, while overwhelmingly affecting adolescent males, can also occur in females and present with the same clinical and imaging features, including orbital and intracranial extension. Clinicians should consider JNA in the differential diagnosis for vascular nasopharyngeal masses, regardless of sex. In extensive disease, thorough cross-sectional imaging, angiographic mapping with selective embolization when appropriate, and coordinated multidisciplinary surgical management are essential. Further reports of female cases, with standardized staging, operative detail, and follow-up, will help clarify potential biological differences and refine management strategies.
